# Embracing an integromic approach to tissue biomarker research in cancer: Perspectives and lessons learned

**DOI:** 10.1093/bib/bbw044

**Published:** 2016-06-01

**Authors:** Gerald Li, Peter Bankhead, Philip D Dunne, Paul G O’Reilly, Jacqueline A James, Manuel Salto-Tellez, Peter W Hamilton, Darragh G McArt

**Affiliations:** Centre for Cancer Research and Cell Biology (CCRCB), Queen’s University Belfast, Belfast, United Kingdom

**Keywords:** integromics, big data, biomarkers, omics, digital pathology, interdisciplinary teamwork

## Abstract

Modern approaches to biomedical research and diagnostics targeted towards precision medicine are generating ‘big data’ across a range of high-throughput experimental and analytical platforms. Integrative analysis of this rich clinical, pathological, molecular and imaging data represents one of the greatest bottlenecks in biomarker discovery research in cancer and other diseases. Following on from the publication of our successful framework for multimodal data amalgamation and integrative analysis, Pathology Integromics in Cancer (PICan), this article will explore the essential elements of assembling an integromics framework from a more detailed perspective. PICan, built around a relational database storing curated multimodal data, is the research tool sitting at the heart of our interdisciplinary efforts to streamline biomarker discovery and validation. While recognizing that every institution has a unique set of priorities and challenges, we will use our experiences with PICan as a case study and starting point, rationalizing the design choices we made within the context of our local infrastructure and specific needs, but also highlighting alternative approaches that may better suit other programmes of research and discovery. Along the way, we stress that integromics is not just a set of tools, but rather a cohesive paradigm for how modern bioinformatics can be enhanced. Successful implementation of an integromics framework is a collaborative team effort that is built with an eye to the future and greatly accelerates the processes of biomarker discovery, validation and translation into clinical practice.

## Introduction

Modern basic scientific research approaches and clinical studies of disease are now demanding the requirement to store, manage and interrogate enormous amounts of information. Hidden among these large, complex, multivariate data are the patterns and clues needed to understand disease processes and drive new methods for diagnosis, prognosis, therapeutics and precision or personalized medicine. Commercial and in-house solutions [1–14] have been developed in an attempt to make the most of this data deluge. Describing this age of big data-driven biomarker research, Weinstein defined integromics as the ‘melding of … diverse types of data from different experimental platforms’ [15, 16], while the American Society for Clinical Oncology uses the term ‘panomics’ to describe similar needs [17].

Given the centrality of pathology to solid tumour analysis, we previously published on our own framework for multimodal data amalgamation and integrative analysis, called Pathology Integromics in Cancer (PICan) [18]. Since then, we have been approached by researchers who want to establish similar systems at their own institutions. This led us to reflect on the process of building PICan and the appropriateness of packaging and distributing PICan as source code. However, the diversity of clinical, experimental and research setups means that an optimal integromics strategy for each institution might be vastly different from the next. Hence, we present here a more detailed discussion on the technical considerations underpinning the formation of an integromics environment to accelerate tissue biomarker research, with a focus on cancer. Key framework decisions were shaped by our local environment, but this article will also discuss some alternative solutions that may better suit other research groups, organizations and multi-institutional collaborations. This perspective is structured around several themes, with reference back to PICan as a case study: integromics overview, database structure, data exchange, multimodal analysis, interdisciplinary collaboration and data control. We conclude with a discussion on the central role integromics will play in the modern era of large-scale multicentre trials and global research consortia [19–21]. We hope that the concepts raised will provide valuable insights from a more detailed technical perspective for institutions and research groups wishing to develop their own integromics framework.

### Precision medicine and integromics for biomarker discovery in cancer

The modern precision medicine approach aims to stratify patients based on specific biomarkers that will inform the most effective treatment for each patient, cutting costs and reducing harm from ineffective treatments [22]. Novel therapeutics target specific cancer-causing mechanisms, inducing fewer side effects but requiring companion biomarker diagnostics, with quick turnaround times, to identify the patients who will benefit most. As multiple patient and tumour characteristics can contribute to a treatment’s effectiveness, this drive towards precision medicine is inevitably spurring an increasing demand for unbiased, automated data collection and analysis across a multitude of high-throughput analytical platforms. The current pace of data generation is far outstripping the ability to analyse these data. Efforts by journals and grant-funding agencies to increase data transparency and encourage data sharing have resulted in large repositories of publicly available data, such as ArrayExpress [23] and the European Nucleotide Archive [24]. The ability to access these databases has increased the interest in integromics or panomics, the integrative analysis of multiple big data types. This is especially true in molecular analyses [7–9, 11, 19, 25–28], where tools such as Partek Genomics Suite (Partek Inc, St Louis, MO, USA), GeneSpring (Agilent Technologies, Santa Clara, CA, USA), tools that harness The Cancer Genome Atlas (TCGA) [25, 26] data portal (e.g. cBioPortal for Cancer Genomics [developed and maintained by the Center for Molecular Oncology and the Computational Biology Center at Memorial Sloan-Kettering Cancer Center] [29, 30], COSMIC [31] and the Broad Institute TCGA GDAC Firehose [32]) and others [33, 34] can facilitate statistical analysis and biological contextualization of omics data. However, in the push towards development of precision medicine, there remains an urgent need for an integromics platform that takes into account non-molecular data. Currently, most of these data types are still held in separate or loosely bound silos, with the onus resting with research groups around the world to seek out these disparate silos and attempt to harmonize the data. Even then, different data types derived from different patient cohorts make it challenging to integrate the data at the patient level. This limits analysis between data types to comparisons of cohort-level summaries, potentially obscuring correlations within the data that may have been apparent if studied at the patient level.

### Integration of tissue phenotype through digital pathology and image analytics

Most molecular analyses on their own seldom consider the histological distribution of biomarkers. This leads to blind spots in our ability to interpret and understand molecular analyses, such as the potential implications of tumour heterogeneity and whether it might correlate with any specific genetic or epigenetic changes. Computer-aided image analysis applied to cancer diagnostics and investigative biomarker research has been in use for several decades [35]. However, the combination of digital pathology with whole slide imaging has truly catapulted the field into the era of big data. Entire stained tissue and tissue microarray (TMA) sections, each potentially comprising hundreds of samples across hundreds of patients, can now be digitized, archived and disseminated without the need to store and distribute the original glass slides [36–38]. Digital image acquisition is now commonplace with multiple established companies, such as Aperio (Leica Biosystems, Nussloch, Germany), Hamamatsu Photonics (Hamamatsu City, Japan), Carl Zeiss (Oberkochen, Germany) and 3D Histech (Budapest, Hungary), offering scanning hardware capable of producing high-resolution digital slide images. Aside from greater ease of collaboration, whole slide imaging unlocks the analytical potential of digital image analysis. Algorithms can be trained to identify tumour regions, delineate margins, classify cellular staining and quantify the results for a wide range of histological phenotypes and cellular markers [39]. Many software packages, including commercial (e.g. PathXL TissueMark, Belfast, UK; Definiens TissueStudio, Munich, Germany; Aperio Genie and Visiopharm Visiomorph, Hoersholm, Denmark), in-house (QuPath, Queen’s University Belfast) and community-driven (e.g. ImageJ [40], Fiji [41], Icy [42] and Ilastik [43]) offerings, now offer access to such algorithms. Computerized image interpretation reduces the subjectivity of manual interpretation [44] and, once properly trained, can batch process a large number of slide images at once. Applied to TMAs, high-throughput tissue interpretation can greatly accelerate tissue biomarker discovery pipelines, especially when coupled with the wealth of molecular data currently becoming increasingly accessible.

In light of this, true integromics today ought to embrace both molecular and digital pathology to uncover and validate a more diverse and robust set of biomarkers that will be key to delivering on the promises of precision medicine [45]. Tissue pathology and histology are essential to the application of more recent molecular methods in solid tumours, required for determining the presence and proportion of tumour cells to ensure sample sufficiency for downstream analysis of nucleic acids and for directing microdissection of tissue samples for molecular analysis [46]. Moreover, tissue context and cellular phenotype are central to understanding tumour heterogeneity and the underlying genomic map of solid tumours. The Immunoscore biomarker [47], for example, applies image analysis to contextualize the interpretation of immunohistochemical staining. The understanding of both how genotype drives phenotype and how phenotypic information influences interpretation of genotype are required for a holistic view of cancer and are therefore essential elements of a modern integromics approach.

## Building large-scale digital resources: PICan overview

**Figure 1:**
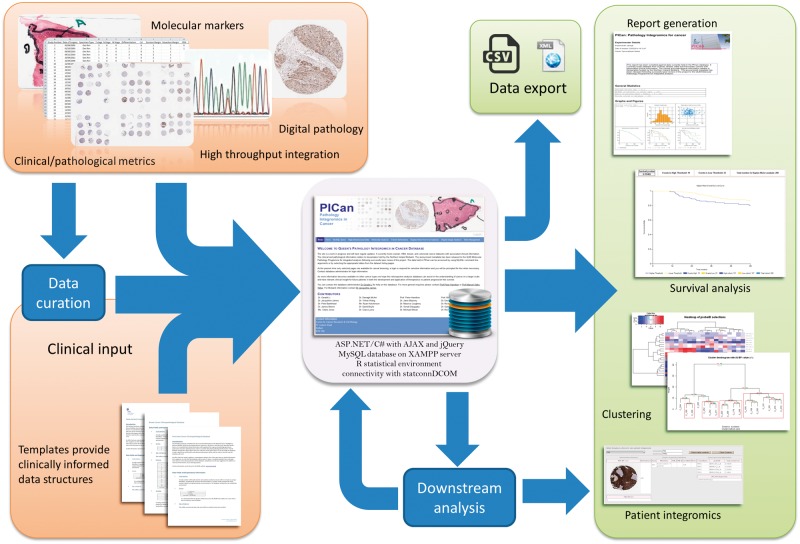
PICan sits at the heart of an integrated multidisciplinary biomarker discovery and validation pipeline. Multiple curated data sources feed into PICan, which accelerates downstream analysis and report generation.

Reflecting this broader view of integromics, we created PICan to be a comprehensive framework for the collection, integration and analysis of clinical, pathological, molecular and digital image analysis data sets for the purposes of cancer biomarker discovery and validation [18]. PICan sits at the heart of our biomarker discovery program, acting as both a central hub and catalyst (Figure 1). PICan streamlines the process of bringing together diverse streams of multimodal data from multiple cancer types to generate clinically and biologically relevant analyses and reports via a bespoke access-controlled web-based interface, written in ASP.NET/C# with a connection to the R statistical environment [48] through statconn [49]. At its core is a restricted-access MySQL database to house this collated patient data. Currently, there are 3660 patients representing eight cancer types recorded in the system together with fully curated data on pathological diagnosis and clinical outcome. From these, nearly 12 000 TMA cores have been stained immunohistochemically and scored for a broad range of well-established and novel cellular biomarkers that are of interest to our basic science, translational and clinical research collaborators. With the ongoing rapid pace of research activity at our institution, these numbers are set to double over the course of the next year. Data de-identification using study-specific unique identifiers protects patient privacy while authentication and authorization of users enables a balance to be struck between enabling access to users and safeguarding the rights of data collectors (clinicians, wet lab scientists and other collaborators) to publication priority and control of their own data. Only administrators have direct access to the PICan database, while end user access is provided exclusively through the access-controlled analysis and report generation web interface. Data quality is a key priority to ensure the integrity of downstream analyses as any analysis is only as good as the weakest data supporting it, so data are curated with biological and clinical input before upload. Meanwhile, bioinformaticians tailor their pipelines according to the needs of the research or analysis. Consequently, in addition to the central database, a complete integromics pipeline must necessarily encompass data collection, data curation and biologically driven analysis and interpretation of this data, activities which we consider integral parts of any integromics framework.

## Database design: data pre-processing, curation and structure

Traditional single-mode data analysis pipelines take raw data (e.g. sequencing reads, microarray spot intensities, whole slide image files) and process it through a series of steps into a final form that informs a particular biological narrative (e.g. list of mutations and frequencies, gene expression values, H-scores for immunohistochemistry (IHC), scoring [50, 51]). However, raw data, such as raw fluorescent spot intensities from an expression microarray experiment, might not be suitable for multimodal and dynamic analysis without corrections for background and batch effects [52, 53], while data aggregation might leave fully processed data, such as a list of differentially expressed genes, too refined to be of further use in patient-level research analysis. A compromise is to capture data in a state that is suitable and informative for subsequent analysis, while tracking metadata associated with acquisition and pre-processing. Metadata, or ‘data about data’, captures information about the methods and conditions under which the data itself were obtained. Such information can be used, for example, to identify cases where an observed effect was the result of differences in the laboratory or researcher conducting the experiments rather than the biological condition. Going back to expression microarray data as an example, this could be achieved by recording background-corrected and normalized probe signal intensities with metadata detailing the nature and parameters for data pre-processing steps such as normalization, assay conditions, instrumentation used, including its settings, and pre-analytical steps, e.g. how the physical samples were obtained, handled and processed and by whom. For completeness and traceability, raw data can be stored alongside this analysis-ready data. Additionally, multiple ‘snapshots’ from single-mode analysis pipelines can be stored as each could potentially serve as a starting point for multimodal analysis. However, care must be taken to ensure internal consistency, so that results are repeatable and reproducible, whether working up raw data or the stored analysis-ready data. Consequently, for PICan, we opted for quality and consistency by recording only the analysis-ready data most suitable for further integromic analysis. A number of quality assurance and quality control measures can be taken to ensure data integrity, with the appropriate level of adoption into standard operating procedures being dependent on the overall level of accreditation of the laboratory in question. Metadata is stored alongside the analysis-ready data, but raw data and most downstream single-mode analysis results are presently excluded from PICan. Future plans to increase data completeness and traceability include adding a central independent repository for raw data files. These represent files not already in existence elsewhere (e.g. public repositories). These raw data files will be read-only and bear unique identifiers that link them to their analysis-ready counterparts on the main database. A file path stored in the main database will allow the raw data files to be easily retrieved when needed. As these files will not be used for on-demand statistical analysis, they can be stored in their native formats.

### Relational database systems

**Figure 2:**
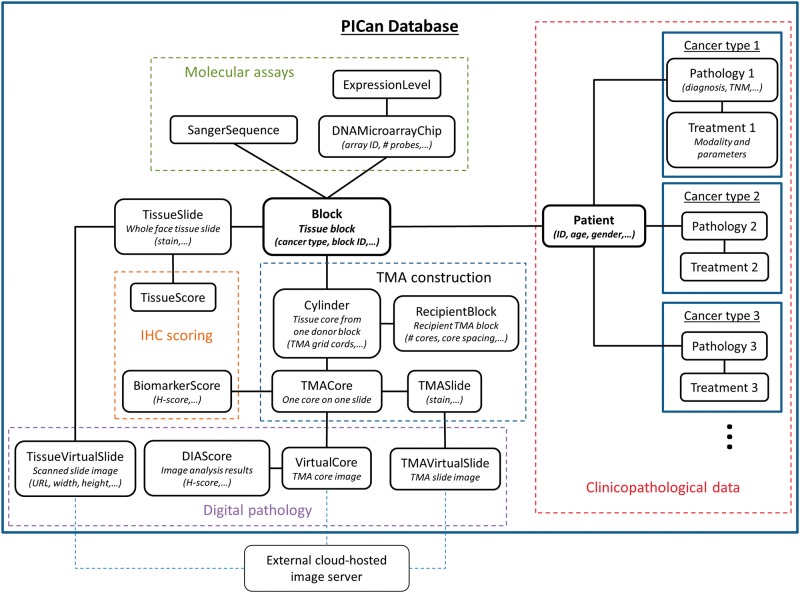
Schematic of the internal table structure of PICan’s relational database, reflecting the core activities and workflows that comprise the biomarker discovery and validation program at our institution. Most tables represent physical or digital entities within these workflows. Where table names may be unclear, a brief description has been included, along with some example data fields (in parentheses). A colour version of this figure is available at BIB online: https://academic.oup.com/bib.

Integromics software infrastructure can be built around one of many database management systems available, each with its own strengths and weaknesses. As we envisioned PICan to incorporate a semi-automated analysis engine [18], we prioritized data quality, which maximizes the validity and reproducibility of our results, and preservation of data structure, which streamlines the automated retrieval and processing of big data sets, rather than attempting to blindly maximize data throughput. Reflecting these design priorities, PICan was built around a relational database (MySQL), consisting of interrelated data tables that reflect the process of constructing and harnessing TMAs, which are central to much of the biomarker discovery work conducted at our institution, the Northern Ireland Molecular Pathology Laboratory and through the Northern Ireland Biobank (NIB) (Figure 2). Every row of every table has a system-generated primary key and these are used as foreign keys to form relationships between tables. The database centres on the *Patient* and *Block* tables (table names will be *italicized*), reflecting our core vision for PICan to drive holistic, multimodal integromic analysis at a patient level. One-to-many relationships link the *Patient* table to tables containing the clinicopathological and treatment data, capturing cancer-type-specific fields from templates developed in collaboration with our clinical expert partners. These templates also define permissible data inputs for each field (e.g. age is a number, sex is ‘M’ or ‘F’), which are then encoded into the database schema. A patient may have had multiple tissue specimens collected as tissue blocks, each represented by a separate linked entry in the *Block* table. Linked via the *Block* table are tables representing molecular assays (*SangerSequence*, *DNAMicroarrayChip*, *ExpressionLevel*), whole face tissue sections (*TissueSlide*), TMA construction (*Cylinder*, *RecipientBlock*, *TMASlide*, *TMACore*), IHC scoring (*TissueScore*, *BiomarkerScore*) and digital pathology (*TissueVirtualSlide*, *TMAVirtualSlide*, *VirtualCore*, *DIAScore*), all of which reflect primary activities within our institution’s biomarker discovery program. Metadata is stored in the table corresponding to the relevant physical entity, and so imaging parameters for a whole face tissue slide, for example, would go in the *TissueVirtualSlide* table. While this database structure is well suited to our needs, laboratories collecting non-tissue specimens such as blood, sputum or urine may find it inappropriate for their needs. A *Blood* table might sit at the heart of a haematological laboratory’s database, for example, while other institutions may need a cluster of tables to represent different specimen types, each linked to tables for various biochemical assays.

PICan uses a modular internal structure to support various data types. Each table represents a physical entity and consequently a data type. This allows the addition of new tables to represent data types from emerging technologies as they mature. As maximizing utility of new tables will require updates to the end-user web interface and setting the appropriate foreign keys will require knowledge of the internal structure of the PICan database, only the system administrator can add new tables.

Another consideration for relational database design is normalization. This procedure simplifies the data structure by removing duplication of data, ensuring that the table is resilient against inconsistencies arising from incomplete addition, deletion or modification of data. As described in [54], this is achieved by identifying groups of related or dependent data fields and splitting them off into a separate table, using foreign keys to record relationships between tables. For most practical applications, the third normal form balances a robust architecture without overly cluttering the schema with an untenable number of tables. Normalization approaches can also be used to optimize database speed and performance.

A consequence of our decision to use a relational database is that free-form data needs to be harmonized with the data structure presented by PICan before data entry. The requisite curation steps prioritize data integrity and prevent poor-quality data that might impede proper and meaningful analysis from entering the database (garbage-in-garbage-out). Clinical and pathological data collection and storage are guided by templates, collections of well-defined data fields that have been developed through our close working relationships with the NIB, Northern Ireland Cancer Registry, Northern Ireland Health and Social Care Trusts and clinicians. These collaborators share the desire to drive data integration and provide expertise, context and data access to harmonize data templates around common vocabulary and permissible data values, ensuring maximal clinical relevance of PICan. In addition to the harmonized common fields like age and sex, PICan also supports cancer-type-specific fields for maximum flexibility.

### Non-relational database structures

While a structured relational database has served PICan well, there are alternative models that might better suit other research integromics environments. MongoDB, for example, is a non-relational (or ‘NoSQL’) database architecture that uses a document model, where each entity (a tissue block, for example) is represented by a ‘document’ that might contain hundreds of attributes as key-value pairs. This model works with a flexible schema, allowing data fields to be added or omitted on a case-by-case basis, while harmonization of terminology and data fields occurs at data retrieval. In a research environment, especially at the beginning of a study when pipelines and metadata are likely to change, it is easier to update the data model than to redesign the fixed schema of a relational database. However, with less urgency to curate data before upload, data integrity may be variable, with errors remaining unnoticed and confounding analyses performed on the data. Fuzzy matching algorithms [55–57] can mitigate the impact of typographical and some clerical errors, but these approaches must still be rigorously validated. In some cases, it might be impossible even for a human expert to deduce the original intention. Data must then be discarded from analysis, potentially reducing the statistical power. Manual curation at the outset might identify such cases when there is still an opportunity to seek clarification from the original data collector. Ideally, ambiguous annotations and borderline cases should be resolved in consultation with a suitably qualified medical professional before they enter the database rather than leaving them open to interpretation by downstream informaticians. Likewise, missing or contradictory data points are preferably identified and, where possible, corrected before entry into the database. However, a major advantage of an unstructured database model is the ability to more faithfully capture the complexities of the patient’s clinical, pathological and other data parameters, including free-form text fields such as clinical notes. The ability to capture patient data in its native form also improves the traceability and scalability of data entry into a non-structured database solution by eliminating the labour and documentation associated with data curation and enabling automated bulk data upload. Despite the tools we have developed to semi-automate data upload, this will remain a relative drawback of manual curation in the short term, especially for clinical and pathological data fields. In the longer term, scalability may be improved with increased adoption of electronic medical records, wherein many of these data fields could be completed by the attending physician when encountering the patient.

Balancing the data curation afforded by the structured relational database with the high-throughput flexibility of unstructured models are various hybrid options. For example, PICan offers flexibility to the structured database model by including freeform text fields to capture unstructured notes (in our experience, these are typically for clinical notes) in several of the data tables. As these fields are not referenced directly by the automated online analysis component, which requires well-defined parameters, they are hidden from analysis but can be retrieved by users wishing to conduct further data refinement or offline analysis. In practice, however, it is more likely that any fields not used in analysis by the system will simply be ignored and neglected. Another hybrid solution we are actively exploring to increase data throughput is to allow the importation of non-curated data sets for end-user analysis. In this model, curated ‘public’ data sets would be available to all users (with necessary approvals and access restrictions in place), while each end user will be able to upload ‘private’ data sets to be analysed alongside or integrated with the curated data. The user, then, is responsible for ensuring the quality of his/her own data. Extending the hybrid model concept further are heterogeneous databases, in which only the most important, unchanging and sensitive data are typically stored in a relational database. Meanwhile, the bulk of the data, in terms of size, will be stored in non-relational databases or as raw data, with links stored in the main database to ensure ease of access and retrieval.

### Image analysis data storage and management

Current work is focussing on improving the support of digital image analysis data, an emerging area of research in which our group has significant expertise. Imaging data presents some unique challenges for database storage design. The images themselves can be large. One of our typical tissue slides scanned at 40× objective magnification (0.25 μm per pixel) can have 2–10 billion pixels. Imaging in RGB at 8 bits per channel gives uncompressed file sizes of up to 30 GB. Additional colour channels (such as for fluorescence imaging), z-stacks and data redundancy in the form of image pyramids for improved image loading performance would all greatly increase file sizes. Compression, typically JPEG or JPEG2000 for whole slide images, can significantly reduce file size without drastically impairing visual assessment, although suitability for image analysis is more variable [39]. Hence, it is vital to store the original slide image to allow a pathologist to go back and verify that image analysis results are consistent with his/her expectations. This is currently achieved by hosting the slide images on remote servers maintained by PathXL. A link is stored in the PICan database that allows the web-based interface to retrieve the image data on user request via an application programming interface (API) supplied by PathXL. Pan, zoom and overlay functionalities in the PICan web interface are powered by OpenSeadragon [58]. As the samples are drawn from NIB collections, NIB maintains control of access and security using the PathXL software.

In addition to the raw image pixels, image analysis data might include markups, annotations and calculated metrics (such as integrated optical densities, nuclear areas or immunohistochemical H-scores [50, 51]). Metrics can be at a cellular level or summarized across a region of interest (ROI), a slide or even a set of slides. Much of these data may be hierarchically structured, with each slide potentially containing multiple ROIs, each ROI containing many cells and each cell being associated with values for numerous metrics. However, the efficiency of storing image analysis data in a structured database is uncertain when a single image can contain thousands of annotations. For now, PICan stores only summary scores and metrics (e.g. H-score [50, 51], Allred Score [59], percentage positive cells), while users wishing to interrogate the image data on a more granular level will need to rely on external specialist tools. The challenge of storing and transferring image analysis data between systems is an ongoing one with several potential approaches having been proposed, including DICOM [60], OME-TIFF [61], various HDF5-based formats [62, 63] and proprietary vendor formats, but without a clear front-runner. DICOM and OME-TIFF are primarily image formats with flexible metadata support, optimized for image viewing rather than storage of image analysis results with large numbers of image objects. In contrast, HDF5 captures object hierarchy relationships well, but on its own it is not a native image format. Meanwhile, proprietary formats are potentially encumbered by patenting and intellectual property concerns.

### Integromics data management is patient-centric

Regardless of the database management system being used, a critical characteristic of integromics analysis is patient-centricity. The ultimate objective in bringing together data from multiple experimental platforms is to build a more holistic picture of the interplay between genotype and phenotype in the patients under study. As interest in amalgamating multiple data types has grown, many platforms originally designed to host one data type have been expanded to accommodate additional types of data. Many biobank management software systems allow addition of clinicopathological notes and experimental assay results on top of sample tracking data. Meanwhile, digital slide hosting platforms like OMERO [64], PathXL Xplore, Cytomine [65] and DigitalScope (Aptia Systems, Houston, TX; used by the College of American Pathologists) [66] are beginning to support the addition of associated clinicopathological data as metadata. Others, like TCGA [25, 26], originated as molecular data repositories but have begun adding whole slide imaging. There is a clear trend towards convergence of database models encompassing more and more data types, but their impact will be limited if these extra data are simply stored and forgotten. Integromics is an active process not just of bringing data together, but more importantly, of creative comparisons and contrasts to expose underlying interactions underpinning the cancer phenotype. Many public repositories and big databases, such as TCGA, draw from an assortment of patient cohorts, so associations between different data types measured on different cohorts are limited to cohort summaries, potentially obscuring trends between data types that are only apparent at the individual patient level.

## Interacting with external resources—data exchange between systems

With the current rapid expansion of big data analytics in biomedicine, integromics frameworks must remain flexible enough to incorporate emerging and future technologies. Structured architecture and design of the overall integromics database framework can facilitate this. However, large data sets like next-generation sequencing (and upcoming third-generation sequencing [67–70]) can be unwieldy in a MySQL database, with typical whole exome or RNA sequencing runs comprising 10–100 billion base pairs for a single sample, so future integromics systems may house these types of data in databases that are structured specifically for this purpose. Instead of a single database housing multiple data types, a future integromics database will undoubtedly be structured as a fully distributed federated database [71–74], with a central database storing identifiers that interface with a cluster of highly specialized databases each housing a specific type of data in its native format. Arguably, PICan already uses a version of this model by outsourcing digital slide image storage to remote servers. Future developments may include enabling PICan to tap into publicly available data sources and allowing users to analyse these public data sets in tandem with PICan data, such as accessing Gene Expression Omnibus data through GEO2R [75].

One of the challenges when working with a federated database model is how to exchange data faithfully and efficiently between the various components. Each constituent database will need an interface to interact seamlessly with the central database and control system. Some of these will be available off-the-shelf or rely on published APIs, while others such as those interacting with in-house software may need significant developer time and effort to implement. Ultimately, there are no shortcuts to establishing these interfaces, and so institutions should aim to reduce the number of distinct interfaces by standardizing data around a small set of formats. For example, when working with whole slide images, a single institution could strive to use only a single file format. The various available formats each have their own strengths and weaknesses, but the natural option will likely be the native format of the scanner used at the institution or a vendor-neutral format like DICOM [60] or OME-TIFF [61]. Fortunately, many tools for working with whole slide images support multiple file formats, such as DigitalScope [66] for remote, web-based viewing of various imaging formats. Meanwhile, projects like Bio-Formats (Open Microscopy Environment consortium) [61] and OpenSlide [76] further facilitate the viewing and exploitation of various imaging formats in other programming and analysis languages like C, C ++, Python and Java. Additionally, it is important to standardize the data transfer protocol whether this is through Internet standards like hypertext transfer protocol, physical means like hard disks or some other agreed protocol. Perhaps establishing an institutional integromics strategy will encourage adoption of standardized data formats by creating an expectation among data generators that data should be portable and compatible with other data types for analysis.

Data flow within an integromics environment can often be multidirectional. Even with only a single central data repository, there will be instances when it is beneficial to export some of these data to a separate third-party platform, whether for specialized analysis, report generation or some other purpose. As PICan’s web-based user interface is designed to be user friendly, it supports only a limited number of types of analyses and report generation options. Analyses not hard-coded into the interface, for example, would need to be performed ‘offline’. In fact, this is how new analyses are tested before being incorporated into PICan. Similar to data import and storage, data export from an integromics system is complicated by the wide range of data types that demand different formats to accommodate their inherently different natures. Efforts have been made in some fields to standardize and integrate data models, such as those of the Global Alliance for Genomics and Health for genomic data [20]. Hence, where widely recognized data exchange formats exist, such as FASTA/FASTQ files for nucleotide or amino acid sequences [77] or the TMA data exchange standard [78], they should be prioritized for integromics support. For other data types like digital image analysis, however, there remains a need for a well-defined and widely accepted standard for data exchange. Whether it is possible or feasible to define a single, unifying integromic data exchange format that would encapsulate multiple data types attached to individuals within a cohort of patient subjects remains an open question.

### Standardization of data vocabulary

Any data exchange standard needs to address both how data are encoded (structure) as well as a common vocabulary for commonly encountered fields. CSV, JSON, XML and derivatives of these are popular formats that lend themselves well to structured data. Certainly, CSV export is our default, as although it does not fully represent the relational structure underpinning PICan’s database, it mimics the basic tabular structure and can be easily viewed by other researchers in common spreadsheet or statistical packages. Non-tabular data like digital slide images (which are stored as links in the database) are exported as separate files. The greater challenge might be agreeing on a common vocabulary. Data field names need to be mapped from PICan’s internal vocabulary to that of the target external platform. This is not a trivial task, as even professionals within the same institution may use different names for the same metric. Some clinicians may be more precise in how certain metrics are measured and recorded, such as when some distinguish between lymphatic and vascular invasion while others group them together as lymphovascular invasion. Harmonizing between cancer types can also be challenging, hence the use of cancer-type-specific tables in PICan for certain clinicopathological metrics. Additionally, even where a data exchange specification exists, like the one for TMA reporting [78], there may be allowance for custom fields, posing an extra challenge for aligning under a common vocabulary. Attempts have been made to standardize terminology in some disciplines. The US National Library of Medicine maintains a collection of controlled vocabularies known as the Unified Medical Language System [79], which incorporates SNOMED CT, MeSH and OMIM, among many other ontologies. Another compendium is caCORE, including the Enterprise Vocabulary Services and Cancer Data Standards Repository of common data elements [80, 81]. Additionally, some groups have devised cancer-type-specific lists, such as for breast [82], mesothelioma [83] and prostate [84]. In the absence of clear community-supported data standards, institutions are advised to agree on and use a minimal set of data export formats.

## Unique challenges of multimodal integrative analysis

Collecting and storing diverse data types is only half the battle in integromics: unlocking the value of these data will come from analysis and interpretation. Through a connection with the R statistical environment via statconn [49], PICan is capable of on-demand statistical analysis, graphical output and report generation. Besides R and statconn, other similar frameworks and interfaces include Shiny [85], R.NET [86], Python (e.g. matplotlib [87]) and D3.js [88]. While PICan is strictly a research tool, an integromic approach will undoubtedly be essential to future clinical decision support systems, placing integromics at the front line of patient care delivery.

Multimodal analysis is particularly important when considering the future of digital pathology and tissue imaging. Conventional light and fluorescence imaging provides the basis for most studies in this field. However, the future may provide new imaging technologies using light outside of the normal visible spectrum that may be more informative towards illuminating the underlying cancer biology and delivering sensitive and specific biomarkers of clinical outcome. Numerous studies are emerging that exploit technologies such as ultraviolet [89], infrared [90] and Raman spectroscopy [91, 92], together with a broad range of associated multispectral technologies, to gain a better insight into tissue and cell characterization and to visualize compounds and chemistry not accessible to conventional microscopy. Integrating these imaging modalities into a single framework to compare, compound and deduce clinical utility, not in isolation, but together with conventional imaging methods is essential. Similarly, there is capacity to integrate other forms of medical imaging relevant to cancer discovery, including positron emission tomography, magnetic resonance imaging, computed tomography and X-ray. This can be used to understand the relationship between *in vivo* clinical imaging and *ex vivo* tissue imaging, thereby translating markers that are currently only visible on microscopy to early *in vivo* detection with clinical imaging modalities. In both settings, the real value is likely to come from the overlay and integration of imaging modalities, extending the capacity of tissue imaging on its own and providing new visualization tools for both pathologists and radiologists that are more informative and consequently improve clinical decision making. The integromics initiative discussed in this article provides such a model for imaging as well as molecular and clinical data, blurring the edges of medical specialties and providing a unified framework for discovery.

The basic tools of integromic data analysis are generally similar to other disciplines of bioinformatics. Mean comparisons, clustering, survival analyses, machine learning and linear models, for example, can all be used, as can the various pipelines that have been developed for next-generation sequencing, gene expression microarrays and other molecular big data omics techniques. Integration of digital image analysis data in integromic analysis can present its own distinct risks and rewards. Images and their associated markups and annotations do not lend themselves on their own to t-tests or clustering, for example. Nevertheless, these may be useful to contextualize other pieces of data like IHC scores. Calculated scores (e.g. H-score) and metrics (e.g. integrated optical density, nuclear area) can often be expressed as name/attribute pairs, so they lend themselves more immediately to integrative analysis, although data structure and hierarchy can still complicate analysis. Some metrics can be measured at a cellular level and summarized at an ROI or whole slide level. Others, however, might not be meaningful at a cellular level (e.g. H-score), so one must be mindful of the meaning of each measure when incorporating it into a multivariate model of the data. Tumour heterogeneity is also an important complication. Summary scores computed across different regions of the same tumour may differ markedly. Heterogeneity, at least in solid tumours, can be more easily mapped in tissue sections where tissue structure and cellular context is maintained and intact. Spatial changes in tumour cell arrangement can be visualized microscopically and used to sample regions for molecular analysis and the measurement of intratumoural heterogeneity. Whole slide imaging and digital image analysis represent powerful tools to quantitatively map tissue phenotype and provide a sound basis for biomarker assay development while opening up new avenues of inquiry. For example, one can begin to explore whether certain morphometric phenotypes correlate with specific genetic or epigenetic profiles and whether these have any significant prognostic or therapeutic impact.

A specific challenge for integromics is the application of statistical techniques to data from disparate sources. Care must be taken to ensure differences observed between experimental groups reflect the underlying biology and not simply batch effects. Differences in experimental assay platforms, such as different probes targeting the same gene or different antibodies for IHC, may produce discrepancies in the data based on different levels of sensitivity and specificity. In digital pathology, differences in imaging hardware, analysis algorithms and parameter definitions may lead to various imaging artefacts and interpretation difficulties. Even within the same software, cell counts determined from different threshold settings are not comparable. Less visible confounders include study and patient variables like recruitment methodology, study admission criteria, patient stratification/diagnostic criteria, pre-collection treatments and standard of care (for prognostic biomarker studies) that can affect the makeup of the study patient population. Meanwhile, specimen handling variables like cold ischemic time, fixation method, staining protocols and formulations can have a profound effect on the data, often dominating other confounders, regardless of the subsequent pipeline. Ideally, then, data harmonization begins well before data are generated: with standardization and thorough documentation of all pre-analytical steps including patient selection, tissue collection and specimen handling. Where potential batch effects or confounding variables have been identified, a number of strategies can be used, such as subset analysis (i.e. stratifying the subject population on the confounding variable) or regression modelling [93, 94].

Mandated public release of large data sets has been helpful in advancing transparency in science, but tracking the associated metadata and assessing its impact on results derived from these big data sets remains a major unresolved challenge. Even large repositories like TCGA are composed of smaller collections from a wide range of geographies and institutional environments. In many cases, pooling such diverse data sets becomes an apples-to-oranges comparison. Batch effects in TCGA and other large high-throughput data sets are well-documented [52, 53]. Without control over data origins and consistency, it is essential that the entire process be rigorously validated before interpretation of results. What local data sets lack in scale, they make up for in traceability. For PICan, we require that our collaborators are able to stand over every data point that enters our system, thereby ensuring a higher standard of quality and confidence in our data. Metadata recording technical and experimental details are stored in PICan alongside the data. Regardless of how it is done, metadata tracking should be rigorous and thorough as differences in experimental conditions and design must factor into the interpretation of any analysis. Where appropriate, data should be corrected and normalized to ensure comparisons are meaningful like-versus-like comparisons and observed contrasts are not merely batch or scalar effects.

## Interdisciplinary teamwork

Apart from robust infrastructure and software support, the heart of any successful integromics framework is a cohesive and interdisciplinary team to support and champion the entire effort. Despite the technical complexities of a system like PICan, teamwork is arguably the most important and yet most often overlooked piece of the integromics puzzle. This is fundamentally a collaborative endeavour, bringing with it all the challenges of working with an interdisciplinary team of individuals and organizations, each of whom have their own diverse set of goals and priorities. Clinical team members, for example, may have responsibilities to their patients that would not apply to academic researchers. Nevertheless, the specialist insights afforded by an interdisciplinary team have been indispensable to the success of PICan. Clinicopathological data tables are built on templates created by clinical experts specializing in each cancer type. Clinical and translational researchers inform the choice of analysis tools to be added, which are then developed and tested by translational bioinformaticians. Establishing and incorporating an integromics framework into the mindset of an institution will require additional effort and workload on certain individuals. Hence, it is imperative that team discussions include delegation and shared responsibility over matters such as data collection and curation, data formats and standards, system maintenance, data exchange and access control. Differing priorities and opinions between team members are to some extent unavoidable, but teams can adopt measures to manage conflicts, such as having all protocols clearly documented or maintaining a regular meeting schedule. Regardless of how these challenges are met, we stress the importance of maintaining open lines of communication and agreeing to clear roles and responsibilities of all team members. Effective teamwork is a non-trivial yet integral contributor to the success of any integromics framework.

With an ever-increasing emphasis on interdisciplinary research, projects like PICan underscore the need for cross-disciplinary education to instil young researchers with the necessary mindset, understanding and skills to navigate the challenges and opportunities in the research environment of the future. Queen’s University Belfast, for example, offers a Masters-level course in Bioinformatics and Computational Genomics [95]. The course attracts students from a diverse range of backgrounds, from medical doctors to engineers and computer scientists to biologists from a range of life science subject areas, and aims to equip them with a solid foundation across a broad range of topics such as advanced bioinformatics, database design, tissue imaging and integromics.

Interdisciplinary teamwork is vital to another pillar of any integromics framework: access to quality data. Any analysis is only as robust as the data that underpins it. The role of the NIB is crucial to PICan’s success not only through access to its biospecimens, but also its expertise and infrastructure support for sample preparation, processing, accessioning, tracking and quality control. The active participation of an interdisciplinary team also distributes the workload of data curation. One of the great struggles of data collection is maximizing quality and quantity with a limited resource of labour. With an increasing focus among funders and institutions on large-scale, multicentre studies, scaling up an integromics framework while preserving data quality will rely heavily on greater delegation of data curation to the data collectors themselves. The onus will be on the individual data collectors to flag data discrepancies, so that the database administrator serves as the last line of defence rather than the first.

## Ethics and data governance

Like any other system handling clinical and pathological information, an integromics framework also requires appropriate approval for the use of patient data. Additionally, data controls in integromics need to protect both patients and data collectors: de-identification protects patient privacy, while access controls and terms of use policies protect the rights of data collectors.

All studies undertaken in PICan need to be approved following an application to NIB, where project-specific ethics and governance are granted under the Office for Research Ethics Committees Northern Ireland approved NIB guidelines. As a research tool, PICan stores no personally identifiable information in its database, ensuring patient anonymization and minimizing the potential impact of any data security breach. Each patient, sample and associated analytical data are labelled using a unique independent study-specific identifier within the system. Clinically qualified and authorized patient data collectors retain the key that links these identifiers to hospital systems where personal data are securely stored. Researchers using PICan are restricted to using anonymized research data only—respecting data protection laws and regulations. However, as important follow-up data on patients accrues over time, these can still be accessed by having appropriately authorized clinical staff and data collectors use the key to identify patients on hospital systems and retrieve more up-to-date patient information. Authorized clinical personnel can then review and retrieve pertinent research-relevant information from the hospital systems and update PICan, again with only anonymized research data using the PICan identifiers. This is equivalent to an honest broker system used elsewhere [96, 97].

Data curation of de-identified information in PICan produces a robust, privacy-controlled snapshot of analytical data, but the underlying data sources are themselves continually being updated as patients are followed up and new specimens are collected. While data update is possible, it is necessarily a manual process as PICan has been walled off from the original data sources by design, allowing it to operate with fewer privacy controls than a typical clinical system, as no identifiable patient data are stored. To maintain quality and consistency with existing data, data updates to PICan are done only periodically, with entire cohorts of patients updated at once. Alternatively, institutions that prioritize constantly updated data will require deeper integration between their research and clinical data systems. In such cases, additional data protection measures will be required, such as encryption or safe havens for data storage and transfer. Essentially, such an integrated system would need to meet local rules and regulations for clinical data systems.

## Scaling up for large-scale epidemiological studies

The last few decades have seen tremendous investment into all aspects of cancer biomarker discovery research, but as of 2013, only 18 protein cancer biomarkers had received US Food and Drug Administration (FDA) approval [98], despite decades of research and thousands of publications on novel candidate biomarkers. Part of the reason may be a focus on early-stage discovery activities and the relative lack of large-scale multicentre validation and clinical trials. Studies of such scale present a number of data management challenges, not unlike those we designed PICan to address. First and foremost, integromics is inherently a team activity and the same principles of teamwork apply whether it is conducted within a single centre or across multiple research centres on different continents. In this way, integromics research extends readily to large-scale multicentre epidemiological studies. Data harmonization of file formats and common vocabulary remain vitally important and best addressed through open communication between collaborators. As in the case of collaborators within an institution, team members from different institutions should ideally agree on a single data format for each data type and a common, agreed set of vocabulary to describe data fields collected, depending on the needs of the study and its participants. Analysis of multicentre data also requires standardization of pre-analytical parameters such as inclusion criteria, sample acquisition, sample processing, assay conditions and measurements recorded. When establishing project standards and protocols, teams should consider what community standards are already available to maximize interoperability and avoid redundancies. Across Europe, for example, projects like ELIXIR [99] and Euro-BioImaging [100] are already seeking to establish hubs for biomedical and bioimaging resource sharing. Globally, similar efforts in genomics are also underway [19–21].

The process of building digital infrastructure to support a multicentre integromics framework is similar to the case of a single institution. All team members should meet and agree on the structure of the framework. Depending on the extent of the project and range of requirements, the technical, scientific and medical team will need to grow as well. Among the team, there is a need for at least one full-time staff member dedicated to technical development and management of the digital infrastructure. As the project grows, the staffing requirement will undoubtedly increase in tandem. For this reason, modular design becomes even more crucial as it allows development tasks to be carved up and delegated out to different team members. There will also be additional requirements for hardware and software licences. Software costs can be mitigated by using free (e.g. R) or low-cost volume licenced (e.g. Microsoft Visual Studio (Redmond, WA, USA) for ASP.NET in an academic setting) options. Nevertheless, despite minor challenges, digital solutions generally scale well both in size and geographic distribution. Parallels can be drawn between incorporating external data resources into an integromics environment, as previously discussed, and the challenges of connecting information systems across multiple sites. However, multicentre teams also face some specific challenges. Teams may need to comply with various sets of local laws and regulations surrounding research ethics, data security and patient privacy. Network access and security may also be further complicated in a multicentre setting. PICan, for example, was built for use within a single institution, so access was limited to within the institution’s network firewall, with all external access blocked. A system spanning multiple institutions would need greater attention to security while maintaining access from outside the host institution. Teams may also need to contend with different software and hardware infrastructures at the various centres and implement the necessary interfaces to ensure compatibility. However, the ultimate key to success of multicentre collaborations is a fundamental commitment to team science and on that front, institutions that embrace integromics will have the experience to lead the coming age of large-scale multicentre studies.

Furthermore, platforms like PICan and the integration of image analysis data with clinicopathological and therapeutic data will help accelerate the screening of candidate tissue biomarkers for prognostic and predictive capacity. While this represents a powerful research and discovery tool, this is likely to identify new tissue biomarkers (based on IHC, chromogenic in situ hybridization (CISH), fluorescence in situ hybridization (FISH), RNAscope, etc.) that have clear value in routine diagnostics and the stratification of patients for precision therapeutics. Further validation of these biomarkers would be necessary in carefully controlled and standardized biomarker trials taking into consideration sample preparation, scanner configuration, lab-to-lab variation, comparative performance against pathologist scoring and clinical outcome data. Regulatory approval of digital diagnostic tests would require FDA clearance (USA) or CE-IVD marking (Europe) if they were to be marketed as clinical tests by industry, but may also be implemented as laboratory-developed tests with documented internal validation and clinical evidence, if done within an academic medical centre such as our own setting. Integromics platforms provide the framework for discovery and if designed appropriately can provide robust clinical evidence of the utility of new biomarkers for subsequent commercial licensing, regulatory approval and clinical translation into routine diagnostic/therapeutic practice.

## Conclusion

Modern biomedical research generates scientific and clinical data faster than the ability to fully interpret it, while data from new and emerging technologies need to be integrated and harmonized with existing analysis workflows. Efforts to resolve the resulting bottleneck in interpretation to deliver on the promises of precision medicine in this era of fast-track biomarker discovery and evaluation have led to the birth of a new field in bioinformatics: integromics. After introducing our own framework for streamlining integromic analyses, it became clear that there is widespread interest in implementing integromic strategies in other research institutions. In light of the present discussion, we feel that an optimal integromics framework must be well-supported by a dynamic and interdisciplinary team, reflect the local needs and priorities of the institution and be designed with an eye to the future. The team is the gateway to high-quality data and specialist insights that inform analysis and ultimately catalyse translation to clinical application in support of precision medicine. Meanwhile, biomarker research at our institution is currently centred on TMAs and we have emphasized data quality and curation over raw throughput. These traits are imprinted in the design and operation of PICan, but other institutions may have other needs that demand a different infrastructure. Ongoing development of PICan includes incorporating raw data storage and access for completeness and traceability and strengthening support for digital image analysis. As our institution adopts newer technologies, PICan likewise will continue to evolve, facilitated by its modular design, to meet the changing needs of a busy cancer research environment spanning numerous programmes, technologies and scientific objectives. Hence, integromics should be interwoven into the fabric of the institution and not simply be a downloadable add-on.

**Key Points**

- Bold approaches are needed to maximize the potential of big data in modern biomedical research and to break down the silos in which they are currently held.
- Collating, storing, transferring and integratively analysing vast amounts of heterogeneous, multimodal data requires a comprehensive and cohesive integromics strategy that streamlines biomarker discovery by exposing fresh insights into the underlying factors contributing to disease phenotypes.
- We explore the practical considerations behind implementing an integromics framework, using our experiences and design choices with PICan as a case study, while recognizing that the global diversity of biomarker discovery research programmes will require tailoring solutions to each research environment.
- Successful integromics frameworks are supported by dynamic interdisciplinary teams and are inherently adaptable to new technologies and the host institution’s evolving priorities.
- Integromics must be a central part of the institutional culture and not just a downloadable add-on.

## Conflict of interest

Professor Peter Hamilton is the founder of, and non-executive director with, PathXL Ltd. Professor Manuel Salto-Tellez is Senior Advisor to PathXL Ltd.

## Funding

The research leading to these results has received funding from the People Programme (Marie Curie Actions) of the European Union’s Seventh Framework Programme FP7/2007–2013/under REA grant agreement (285910 to P.W.H.); Invest Northern Ireland (RDO0712612 to P.W.H.); Cancer Research UK Accelerator (C11512/A20256 to P.W.H./M.S.-T.); CRUK (to P.B.). The Northern Ireland Molecular Pathology Laboratory is supported by Cancer Research UK, Experimental Cancer Medicine Centre Network, the NI Health and Social Care Research and Development Division, the Sean Crummey Memorial Fund, the Tom Simms Memorial Fund and the Friends of the Cancer Centre (to M.S.-T.). The Northern Ireland Biobank is funded by the Health and Social Care Research and Development Division of the Public Health Agency in Northern Ireland and Cancer Research UK through the Belfast CRUK Centre and Northern Ireland Experimental Cancer Medicine Centre; additional support was received from the Friends of the Cancer Centre (to J.A.J.).

## Notes

**Gerald Li** is a Research Fellow at Queen’s University Belfast. His research focuses on integrative multimodal data analysis for cancer biomarker discovery and validation.

## Notes

**Peter Bankhead** is a Research Fellow at Queen’s University Belfast. His research focuses on developing novel image analysis algorithms and software, focussing on digital pathology.

## Notes

**Philip Dunne** is a Senior Research Fellow at Queen’s University Belfast. His research has evolved from basic biology to using translational bioinformatics to define the molecular pathology epidemiology of early-invasive cancer.

## Notes

**Paul O’Reilly** is a Research Fellow at Queen’s University Belfast. His research focuses on the development, implementation and acceleration of bioinformatics algorithms, and the architecture of bioinformatics software systems.

## Notes

**Jacqueline James** is a Clinical Senior Lecturer/Consultant Pathologist at Queen’s University Belfast/Belfast Health and Social Care Trust. Her research uses integrative molecular approaches for biomarker discovery and validation.

## Notes

**Manuel Salto-Tellez** is the Chair of Pathology at Queen’s University Belfast and a diagnostic morpho-molecular pathologist. He has championed the integration of phenotype and genotype in diagnostics and research.

## Notes

**Peter Hamilton** is Professor of Bioimaging and Informatics at Queen’s University Belfast. He has been working for the past 25 years in digital pathology, quantitative imaging, biomarker discovery and digital diagnostics.

## Notes

**Darragh McArt** is a Lecturer in Translational Bioinformatics at Queen’s University Belfast. His research focuses on developing accelerative and integrative solutions in modern biomarker research.
